# NRF2 and mutant p53 separately regulate redox balance in esophageal squamous cell carcinoma through metabolic reprogramming

**DOI:** 10.1080/15384047.2026.2745287

**Published:** 2026-11-06

**Authors:** Guodong Deng, Yue Liu, Wei Zheng, Lili Qiao, Lei Xu, Yanan Yang, Hui Yuan, Xiaohong Yu, Wei Jiang, Xiying Yu

**Affiliations:** a Department of Etiology and Carcinogenesis and State Key Laboratory of Molecular Oncology, National Cancer Center/National Clinical Research Center for Cancer/Cancer Hospital, Chinese Academy of Medical Sciences and Peking Union Medical College, Beijing, China; b State Key Laboratory of Molecular Oncology, National Cancer Center/National Clinical Research Center for Cancer/Cancer Hospital, Chinese Academy of Medical Sciences and Peking Union Medical College, Beijing, China; c Beijing Key Laboratory for Carcinogenesis and Cancer Prevention, National Cancer Center/National Clinical Research Center for Cancer/Cancer Hospital, Chinese Academy of Medical Sciences and Peking Union Medical College, Beijing, China

**Keywords:** ESCC, NRF2, p53, metabolic reprogramming, redox balance

## Abstract

**Background:**

Alterations in CUL3-KEAP1-NFE2L2 (CKN) axis and p53 lead to abnormal NRF2 activation and p53 dominant-negative (p53DN) effects to perturb the redox state in esophageal squamous cell carcinoma (ESCC). The precise roles of these, especially their interplay, in these processes remain controversial and elusive.

**Methods:**

We investigated the genetic alterations of the CKN axis and p53 in ESCC/SCCs and their interplays via bioinformatical analysis and immunoblots. Specific downstream target(s) of them were identified using bulk-RNA sequencing, RT-qPCR, immunofluorescence, and immunoblots in the immortalized esophageal cell, D3. The effects of redox balance in ESCC chemoprevention were performed using an NMBzA-induced rat ESCC model.

**Results:**

NRF2 and p53DN (p53^R246W^) enhanced the antioxidant capacity. The pentose phosphate pathway and glutathione metabolism reprogramming were responsible for NRF2-related antioxidant capacity, with G6PD and SLC7A11 were critical targets. However, no direct interplay between NRF2 and p53 was detected. The EGFR-ERK-ETS1-SLC7A11 axis is potentially responsible for the antioxidant potency of p53^R246W^-D3 cells. Interestingly, the exogenous oxidant artesunate could suppress carcinogenesis in the rat ESCC model.

**Conclusions:**

Abnormal NRF2 activation and the effects of p53DN appear to dysregulate the redox balance independently during ESCC carcinogenesis, suggesting that targeting the redox balance could be a promising and effective ESCC prevention and treatment strategy.

## Introduction

Esophageal cancer (EC) is one of the most lethal malignancies of the upper digestive tract, with high morbidity and mortality worldwide. Squamous cell carcinoma (ESCC) is the predominant histologic type of EC, with a high prevalence in Northeast Asia and South Africa.^1^ ^,^ ^2^ The etiological causes of ESCC are diverse, including exposure to carcinogens, poor diet, mechanical injury, inflammatory stress, and many other unpredictable factors that directly/indirectly lead to this disease with complex genomic/epigenomic alterations. To date, the available precision medicine strategies for ESCC are dismal, with 5-year overall survival rates of less than 40%.^3^ ^,^ ^4^ Therefore, pathogenesis studies and therapeutic target development for ESCC are urgently needed.

The redox balance/redox homeostasis is required for cell viability in both normal and tumor cells. Under physiological conditions, cells maintain a more reductive intracellular environment than in the extracellular space, which allows them to function well and have a high buffering capacity against oxidants.^5^ As a steady redox state, this homeostasis is tightly controlled by antioxidant systems via the concentration of reactive oxygen species (ROS, a general term for many types of endogenous oxidant molecules). Physiologically, an appropriately elevated ROS level causes “oxidative stress”, which inhibits the progression of the cell division cycle, promotes cell deoxidation and/or repair of oxidative damage, and is ultimately beneficial for cell survival and subsequent proliferation.^6^ However, during the process of carcinogenesis, numerous mitogenic/oncogenic signals lead to a high metabolic state, followed by accelerated ROS production.^7^ Thus, the redundant ROS will lead to detrimental “oxidative distress” and give rise to irreparable “bio-genotoxicity”, impeding the tumor progression process.^8^ Under these conditions, tumor cells have a low buffering capacity and are vulnerable to endogenous and exogenous oxidative stimuli.^9^

Accumulating evidence indicates that the CUL3-KEAP1-NFE2L2 (CKN) axis and the TP53 (p53) pathway function as two of the major redox-regulatory pathways in mammals.^10^ ^,^ ^11^ While the nuclear factor erythroid 2-related factor 2 (NRF2), encoded by the NFE2L2 gene, is a key antioxidant transcription factor whose protein stability is negatively regulated by the E3 ubiquitin ligase CUL3-KEAP1 via 26S proteasome degradation under unstressed conditions, p53 also plays an important role in maintaining the redox balance/homeostasis associated with cellular metabolism in addition to its critical function in controlling genome stability.^10^ ^,^ ^12-14^ For instance, upon exposure to stress, KEAP1 loses its ability to ubiquitinate NRF2, resulting in nuclear accumulation of NRF2, which activates a set of cytoprotective genes. Furthermore, p53 can also inhibit the expression of SLC7A11, an important solute protein antiporter, that is involved in the regulation of glutathione (GSH) synthesis, thereby inducing oxidative ferroptosis.^15^

In ESCC, mutations in the p53 gene are detected in > 90% of tumor samples and more than 10% of the genes in the CKN axis are mutated, with the mutation patterns remaining mutually exclusive.^16-18^ Furthermore, our recent study showed that 52.8% of pathological specimens from ESCC patients without NFE2L2 gene mutations displayed nuclear localization of NRF2, suggesting that NRF2 activation is also regulated by overexpression and/or post-translational modifications in addition to genetic alterations.^19^ ^,^ ^20^ However, the functional roles of mutated and/or overexpressed NRF2 proteins involved in ESCC carcinogenesis remain controversial.^21^ While some studies revealed that abnormal nuclear accumulation and activation of NRF2 could directly/indirectly target hundreds of NRF2 downstream targets to exert complicated antioxidant functions through various pathways, such as direct free radical detoxification, GSH anabolism, glycolysis, etc. to promote ESCC carcinogenesis, other studies showed that the activation/nuclear accumulation of NRF2 resulted in ESCC tumor suppression.^7^ ^,^ ^16^ ^,^ ^22-24^ Moreover, p53 mutations can also directly cause increased ROS levels through AMPK/mTOR signaling, although the specific mechanisms by which p53 is involved in controlling the redox balance are much more complex and not fully understood.^25^ In addition, NRF2 has been reported to be involved in redox regulation with p53 in several studies, but the results also seemed to be contradictory. Liu et al. showed that mutant p53 could associate with NRF2 to downregulate the expression of downstream factors, thereby exerting pro-oxidative effects, while Lisek et al. revealed that mutant p53 could enhance the NRF2 nuclear localization on ARE and initiate the transcription process, thereby mediating antioxidative effects.^26^ ^,^ ^27^ Nevertheless, given the fact that the CKN axis and the p53 pathway are frequently mutated in ESCC, resulting in the disruption of cellular redox balance/homeostasis with increased levels of oxidative stress, targeting the critical redox balance/homeostasis regulators to kill tumor cells may be a promising ESCC treatment strategy.

Previous studies have demonstrated that GSH and NADPH are both important physical substrates for antioxidant effects and that tumor cells have a low buffering capacity that is pro-oxidative and/or susceptible to endogenous and exogenous oxidative stimuli.^28-30^ Therefore, we and others propose that appropriate manipulation of GSH and NADPH involved in redox balance/homeostasis should enhance oxidative damage in tumor cells. More effectively than in normal cells, postulating an important strategy for cancer prevention and treatment.^19^ ^,^ ^31^ GSH exerts a direct antioxidant effect under the catalysis of GSH peroxidase (GSHPx), while NADPH drives the reduction process of oxidative GSH and functions as a critical reducing cofactor for GSH reductase (GSR).^28^ Under endogenous or exogenous oxidative stress, NRF2 activation enhances both GSH and NADPH synthesis by upregulating SLC7A11 and G6PD expression. SLC7A11 initiates intracellular cysteine transport and G6PD mediates the shunting of glycolysis to the pentose phosphate pathway (PPP), both of which are rate-limiting for PPP and GSH anabolism.^23^ ^,^ ^32^ These supply-and-demand processes relieve cellular oxidative pressure, which is consistent with the aforementioned hypothesis that targeting the critical regulators of redox balance/homeostasis, i.e. SLC7A11 or G6PD, is a promising cancer treatment strategy. However, this hypothesis has not been tested in ESCC.

In this study, we first detail the genetic alterations of the CKN axis and p53 in ESCC and examine the NRF2-dependent transcriptome changes, aiming to define whether a downstream target(s) of NRF2, specifically SLC7A11 or G6PD, may serve as one of the critical regulators of the redox balance/homeostasis required for oxidative stress/cell death in ESCC cells, particularly ESCC cells harboring the aberrant CKN axis. We then examine the regulation of oxidative stress in non-transformed esophageal keratinocytes and ESCC cell lines with different CKN and p53 gene backgrounds. Finally, we investigated the effects of oxidative stress involved in ESCC carcinogenesis in vivo using an NMBzA (*N*-nitroso-*N*-methylbenzylamine)-induced rat ESCC model treated with artesunate, an oxidant derived from natural artemisinin. Our results demonstrate that NRF2 and p53 regulate the redox balance/homeostasis separately during ESCC carcinogenesis, but both do so via the SLC7A11-dependent metabolic mechanism.

## Results

### Frequent genetic alterations in the CKN axis in human ESCC and upregulation of antioxidant capacity in esophageal squamous epithelial cells

Previously, we and others revealed that genetic alterations of the CKN axis were found in human ESCC, but the alteration frequencies of each individual component of the CKN axis and their roles in ESCC carcinogenesis remained controversial.^16^ ^,^ ^19^ ^,^ ^33^ ^,^ ^34^ Therefore, we decided to revisit this issue and analyzed the ESCC genomic data available from TCGA. Our results indicated that, among 317 human ESCC samples examined, the genetic alteration rates in the CUL3, KEAP1, and NFE2L2 genes were 4%, 4%, and 9%, respectively, consistent with previous reports. Notably, these genetic alterations in each individual component of the CKN axis displayed a mutually exclusive pattern, which was also consistent with previously reported results (Figure S1A and S1C).^16^ ^,^ ^19^ ^,^ ^35^ We also extended our analysis to other TCGA human squamous cell carcinoma (SCC) data, including human head and neck squamous cell carcinoma (HNSCC, *n* = 640) and lung squamous cell carcinoma (LSCC, *n* = 1065). We found that LSCC had the highest rate of genetic alterations in the CKN axis genes (25%), followed by ESCC (17%) and HNSCC (16%). Taken together, the corresponding genetic alteration rates of the CUL3, KEAP1, and NFE2L2 genes in human SCC were 5%, 7%, and 12%, respectively (Figure S1B and S1C). Like the results obtained in ESCC, the genetic alterations of the CUL3, KEAP1, and NFE2L2 genes in LSCC and HNSCC also displayed the mutually exclusive pattern. These results argued that the protein products of the CUL3, KEAP1, and NFE2L2 genes functioned as the CKN axis to modulate NRF2 nuclear localization and transcriptional activity that controlled the cellular oxidative balance/homeostasis, as indicated by our study and other previous studies. Thus, we hypothesized that the genetic alterations of the CKN axis could perturb the cellular oxidative balance/homeostasis, through leading to the metabolic reprogramming mechanism involved in human SCC carcinogenesis.

To test this hypothesis, we analyzed the genetic alterations in each individual gene of the CKN axis in SCC in detail. We found that the missense and deletion mutations were predominantly observed in CUL3. Although the mutations were detected throughout the entire length of the protein, a hot spot site was found in the CUL3 Nedd8 domain. Since CUL3 functioned as a scaffold protein in the E3 ubiquitin ligase to degrade NRF2, its inactivation resulted in the stabilization/activation of the NRF2 protein (Figure S1D).^36^ These results suggested that these mutations in the CUL3 gene were the loss-of-function (LOF) mutations, consistent with a recent report showing that LOF variants in the CUL3 gene caused a syndromic neurodevelopmental disorder.^37^ Similarly, mutations found in KEAP1, an adaptor protein required for the interaction of NRF2/E3 ligase for NRF2 degradation, were mainly located in the BTB domain (CUL3 combination and dimerization), the BACK domain (oxidation sensing) and the Kelch domains (NRF2 recognition) of KEAP1, the inactivation of which also resulted in the stabilization/activation of the NRF2 protein (Figure S1E).^20^ Thus, mutations in the KEAP1 gene resulted in LOF of the gene product, KEAP1. In contrast, the mutations in the NRF2 proteins were mainly observed in the *N*-terminus of the NRF2 protein, where the amino acids 16–86, containing 7 important ubiquitination sites and 2 known ^29^DLG^31^ and ^79^ETGE^82^ motifs for KEAP1 interaction, were frequently mutated, resulting in the disruption of the degradation process of NRF2 and inducing NRF2 accumulation in the cytoplasm (Figure S1F).^20^ ^,^ ^38^ ^,^ ^39^ These mutations in the NFE2L2 gene caused aberrant activation of the gene product (gain-of-function, GOF), NRF2. Taken together, these results supported the notion that the genetic alterations in the CKN axis disrupted KEAP1-CUL3-mediated NRF2 degradation, resulting in the accumulation of NRF2 protein, including NRF2 nuclear localization and transcriptional activation in human stratified squamous epithelial cells, thereby perturbing the cellular redox balance/homeostasis and ultimately participating in SCC carcinogenesis.

To ascertain that the genetic alterations in the CKN axis resulted in NRF2 activation, we examined the subcellular localization of NRF2 in ESCC cell lines with different CKN genetic backgrounds using immunofluorescence analysis. As shown in Figure 1A, NRF2 was mainly localized in the nuclei of the KYSE 70 and KYSE 180 cell lines harboring with NRF2 mutations (NRF2mut), as opposed to the KYSE 150 and KYSE 450 cell lines, which retained the wild-type NRF2 (NRF2wt) and displayed NRF2 cytoplasmic localization. Consistently, transcriptome analysis using TCGA data further revealed that ESCC samples with the CKN axis mutations expressed high mRNA levels of NRF2 downstream genes NQO1, ME1, PGD, TALDO1, GCLC, and GCLM than ESCC samples without the CKN axis mutations (Figure S2A–B). Moreover, we treated the NRF2wt cell lines, KYSE 150 and KYSE 450, and an hTERT-immortalized rat “normal” non-transformed esophageal keratinocyte cell line, D3, which was established in our lab, with the high-affinity small molecule, KI696.^40^ ^,^ ^41^ This molecule blocks the interaction of NRF2 with KEAP1. As shown in Figure 1B-D, immunoblotting revealed that D3 cells treated with KI696 exhibited significant nuclear accumulation of NRF2 compared with untreated control cells. Notably, KI696-induced NRF2 nuclear translocation and accumulation in KYSE150, KYSE450, and D3 cells resulted in the upregulation of NQO1, an NRF2 downstream target. These results indicated that the nuclear localization of NRF2 depended on CKN axis activation, which increased NRF2 transcriptional activity in esophageal squamous epithelial cells. The mutations in CKN axis genes in ESCC cells resulted in nuclear NRF2 accumulation and the activation of NRF2 transcription.

**Figure 1.**
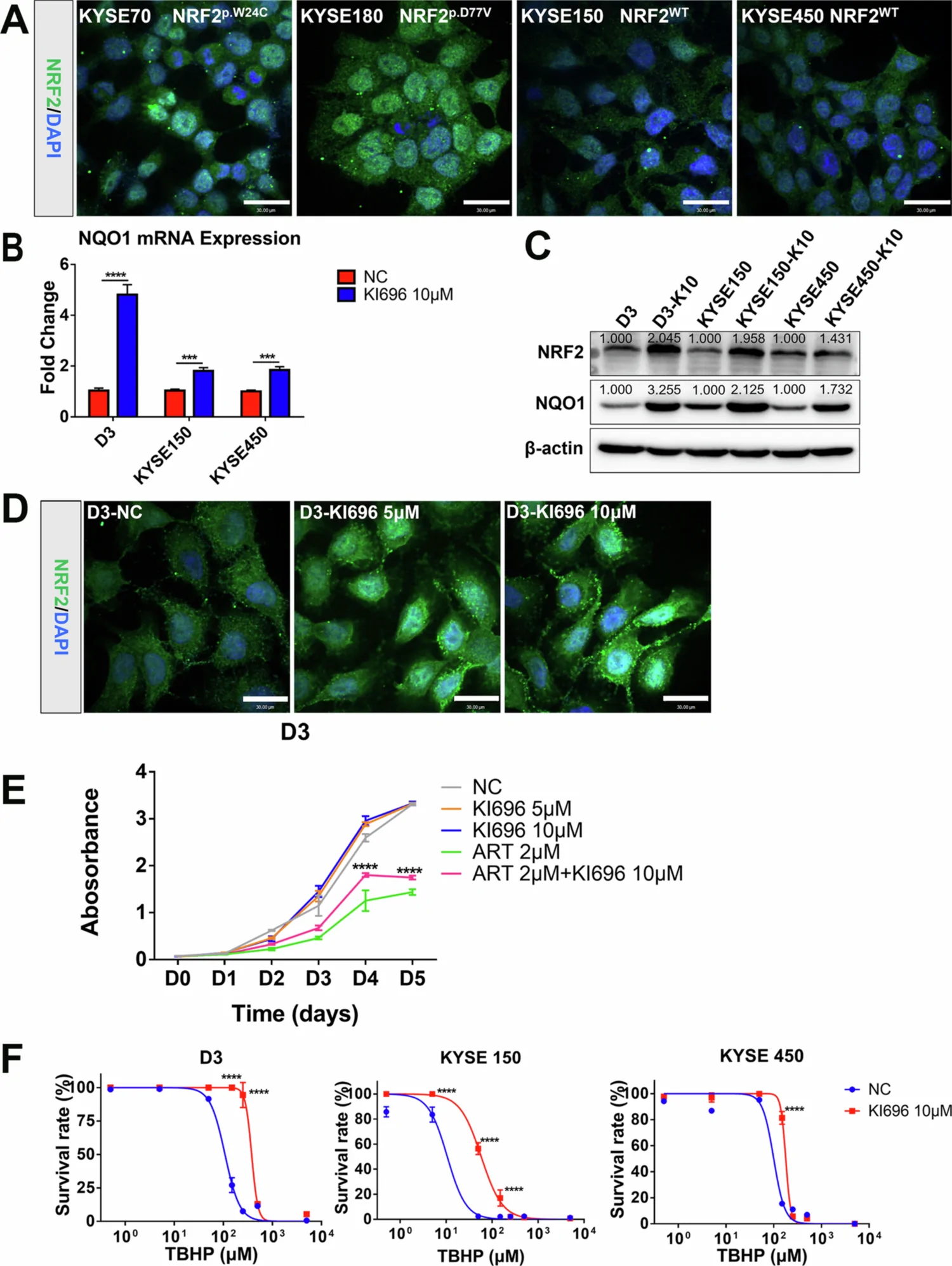
NRF2 localization in cell lines with different gene backgrounds and its anti-oxidation capacity. (A) Immunofluorescence staining of NRF2 localization in NRF2-mutated cell line KYSE 70 and KYSE 180, and in NRF2 wild-type cell line KYSE 150 and KYSE 450. Scale bar 30 μm. (B, C) RT-qPCR and immunoblot of NRF2 downstream NQO1 expression change after KI696 treatment at 10  μM in NRF2 wild-type cell line KYSE 150 and KYSE 450. (D) Immunofluorescence staining of NRF2 nuclei translocation after KI696 treatment at 5  μM and 10  μM in D3 cells. Scale bar 30 μm. (E) Cell viability change of D3 cells treated with KI696 and/or ART. (F) D3 cell death induced by TBHP treatment at gradient concentration and the rescue effect of KI696 treatment at 10  μM. *p < 0.05, **p < 0.01, ***p < 0.001, *****p < 0.0001. ART, artesunate.

However, we noted that D3 cells treated with KI696 did not increase their capacity for cell proliferation, contrary to the results of a previous study (Figure 1E).^42^ These results indicated that the nuclear accumulation and activation of NRF2 alone did not enhance cell cycle progression in D3 keratinocytes in vitro. Therefore, we determined whether the nuclear accumulation and activation of NRF2 were mainly involved in the regulation of the cellular oxidative balance status in D3 cells. We exposed KYSE 150, KYSE450, and D3 cells to tert-butyl hydroperoxide (TBHP), a known inducer of oxidative stress and cellular damage in cells that acted at least in part by depleting the antioxidant glutathione, alone or together with KI696 (for details, see Materials and Methods). As shown in Figure 1F, TBHP could sufficiently kill KYSE 150, KYSE 450, and D3 cells at a micromolar level. However, KYSE 150, KYSE 450, and D3 cells displayed significant improvements in cell viability after co-treatment of TBHP with 10 μM KI696. Similar results were also obtained when D3 cells were treated with another oxidation inducer, artesunate (ART),^43^ alone or together with KI696. As shown in Figure 1E and Figure S2C–D, while treatment of D3 cells with ART alone significantly increased D3 cellular ROS levels and inhibited cell viability, treatment of D3 cells with ART and KI696 could rescue the adverse effects of ART treatment alone.

In sum, the results obtained in this section demonstrated that frequent genetic alterations in the CKN axis were detected in human ESCC, resulting in the nuclear accumulation/activation of NRF2 protein in ESCC cells. NRF2 functioned as a transcription factor to upregulate NRF2 downstream targets involved in the regulation of cellular oxidative balance, thereby increasing the antioxidant capacity, and promoting cell viability in esophageal squamous epithelial cells under the oxidative stress.

### Simultaneous regulation of glycolysis metabolism and GSH anabolism by NRF2 in esophageal squamous epithelial cells

We next sought to elucidate the biological processes of the NRF2-dependent antioxidant effects in esophageal squamous epithelial cells and to identify critical NRF2 downstream targets that could serve as potential biomarkers for ESCC diagnosis and/or treatment. To this end, we performed bulk RNA sequencing (bRNA-seq) in D3 cells treated with and without 10 μM KI696. As shown in Figure 2A, 242 genes were upregulated, and 158 genes were downregulated in KI696-treated D3 cells compared to control KI696-untreated D3 cells. Notably, the KEGG analysis revealed that KI696 treatment in D3 cells significantly induced the activation of the pentose phosphate pathway (PPP) and the GSH anabolism pathway, which are responsible for the production of GSH and NADPH, respectively (Figure 2B). Given that KI696 induced the nuclear accumulation/activation of NRF2 in D3 cells and that the PPP and the GSH pathways were both critical for the intracellular antioxidant material synthesis and ROS homeostasis,^28^ ^,^ ^29^ we hypothesized that these two pathways could work together in the NRF2-induced antioxidant biological processes.

**Figure 2.**
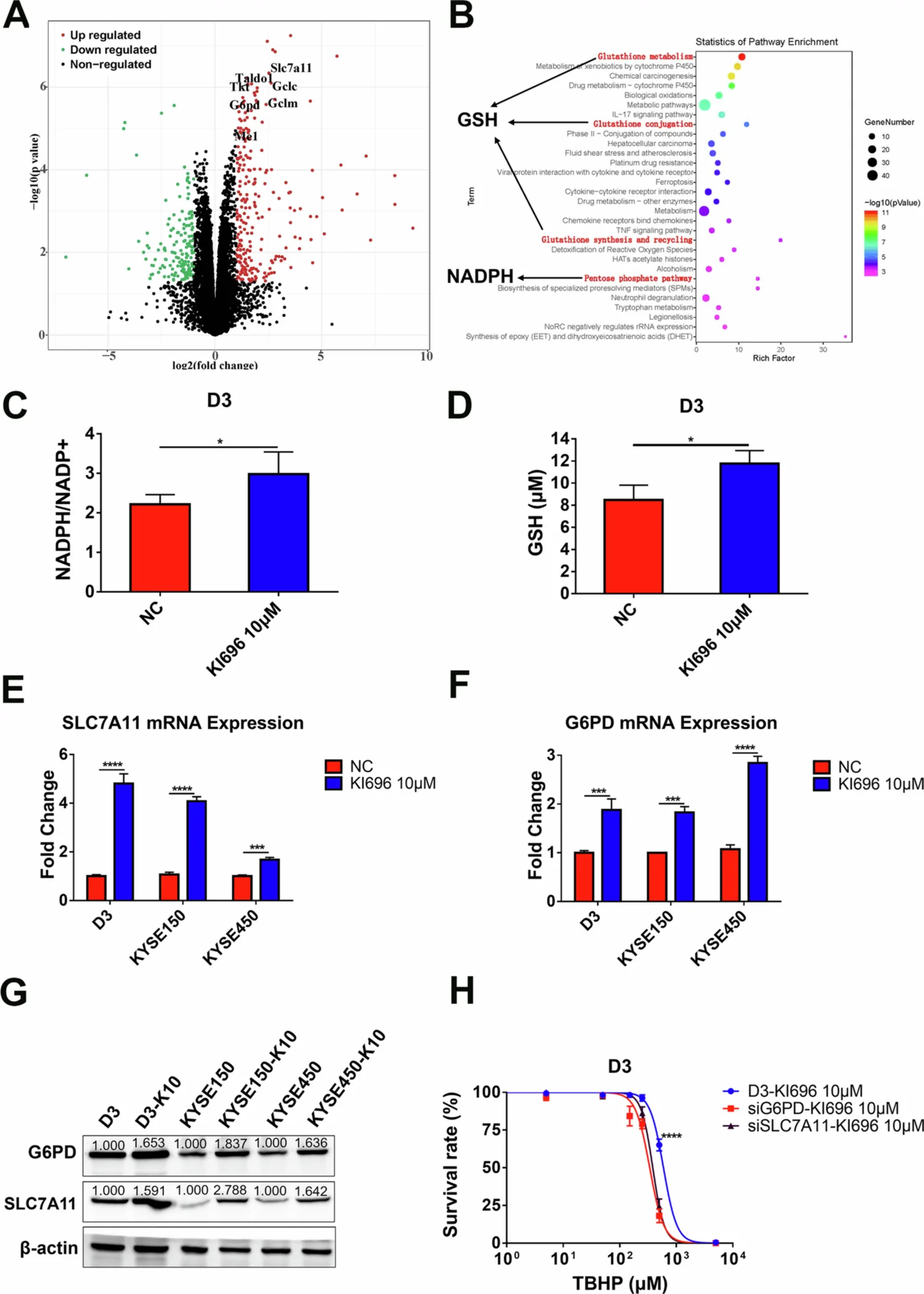
NRF2 regulates glycolysis metabolism and GSH anabolism in esophageal squamous epithelial cells simultaneously. (A) Volcano plot of bulk RNA sequencing in D3 cells treated with and without KI696 at 10 μM. The significantly up-regulated genes are labeled. (B) KEGG analysis shows enrichment of genes of the pentose phosphate pathway (labeled as NADPH) and the GSH anabolism pathway (labeled as GSH) in D3 cells after KI696 treatment. (C, D) NADPH/NADP+ and GSH level changes in D3 cells treated with KI696 at 10 μM. (E–G) RT-qPCR and immunoblot of G6PD and SLC7A11 changes after KI696 treatment at 10 μM in D3 cells. K10, KI696 10  μM. (H) D3 cell viability changes under TBHP treatment at gradient concentration in the condition that G6pd and Slc7a11 are knocked down using RNAi. *p < 0.05, ***p < 0.001, *****p < 0.0001.

To test our hypothesis, we first determined whether the NADPH/NADP + levels and the GSH levels were increased in D3 cells treated with 10 μM KI696. As shown in Figure 2C-D, NADPH/NADP+ and GSSG/GSH assays revealed that KI696-treated D3 cells had significantly increased their intracellular levels of both NADPH/NADP+ and GSH compared to untreated control D3 cells, indicating that both NADPH/NADP+ and GSH pathways were upregulated by NRF2 in D3 cells upon KI696 treatment. Next, we identified the upregulated gene expression of the components involved in the NADPH/NADP+ and the GSH pathways from the D3 bRNA-seq data. Among the list of top upregulated genes, G6pd, Tkt, and Taldo1 in the PPP and Slc7a11, Gclc, and Gclm in the GSH anabolism were identified, which was also consistent with our transcriptome analysis using TCGA data (Figure 2A and Figure S2A–B). Given that G6PD was the key enzyme that played a shunting role as the turning point required to redirect glucose-6-phosphate from glycolysis to PPP to increase the production of NADPH,^23^ we selected G6PD as one of the NRF2 downstream targets for further study. Meanwhile, we noted that the intercellular cysteine was required for GSH anabolism, the synthesis of which was mainly produced from extracellular cystine imported by the antiporter SLC7A11 that mediated the synchronous 1:1 export of glutamine.^44^ Therefore, we also selected SLC7A11 as another downstream target of NRF2 for further investigation. RT-qPCR and immunoblotting showed that the mRNA and protein levels of G6PD and SLC7A11 were markedly increased in KI696-treated KYSE150, KYSE450, and D3 cells compared to those of the KI696-untreated controls (Figure 2E–G). These results were consistent with previous reports that G6PD and SLC7A11 were direct downstream targets of NRF2 using chromatin-immunoprecipitation (ChIP) assay (Figure S3A–B).^32^ ^,^ ^45^ Similar results were also obtained from our transcriptome analysis by TCGA data, which showed that high expression of G6PD and SLC7A11 was present in CKN axis mutant ESCC/SCC samples (Figure 4E–G). Finally, we examined whether G6PD or SLC7A11 was necessary for the NRF2-dependent antioxidant effects. To do so, we depleted G6PD or SLC7A11 expression in D3 cells using RNA interference (RNAi) (Figure S3C–D). As shown in Figure 2H, depleting G6PD or SLC7A11 resulted in decreased antioxidant effects in KI696-treated D3 cells. Taken together, these results indicated that the treatment with KI696, which induced the NRF2-dependent antioxidant effects, could be suppressed by depleting key enzymes involved in glycolysis metabolism and GSH anabolism, such as G6PD and SLC7A11. Taken together, these results indicated that the nuclear localization/activation of NRF2 was simultaneously involved in regulating glycolysis metabolism and GSH anabolism in esophageal squamous epithelial cells. Downstream targets of NRF2, G6PD, and SLC7A11 may serve as biomarkers for the diagnosis and/or treatment of ESCC.

### Demonstration of both G6PD and SLC7A11 as pro-oxidative therapeutic targets in ESCC cells with the aberrant CKN axis

We examined whether G6PD and SLC7A11 could serve as potential pro-oxidative therapeutic targets in ESCC cells with an aberrant CKN axis. Therefore, we administered the G6PD-specific inhibitor RRx001, and the SLC7A11-specific inhibitor HG106 to four ESCC cell lines with different NRF2 genetic backgrounds: KYSE70, KYSE180, KYSE150, and KYSE450.^46^ ^,^ ^47^ As shown in Figure 3A-B, administering both RRx001 and HG106 to ESCC cell lines resulted in cell death. However, the efficacy of RRx001 and HG106 in killing cells varied depending on the NRF2 genetic background of the cells. The IC50 of RRx001 was 5.65 μM in KYSE70 cells and 14.56  μM in KYSE180 cells (both harboring NRF2 mutations), and 17.18 μM in KYSE150 cells and 27.18 μM in KYSE450 cells (both having the wild-type NRF2 genetic background). These results indicated that ESCC cell lines with the NRF2mut gene background were more sensitive to RRx001 treatment than were those with the NRF2wt gene background, as expected. Similar results were observed in ESCC cell lines treated with HG106. The IC50 of HG106 was 4.64 μM in KYSE70 cells and 4.15 μM in KYSE180 cells, both of which harbor NRF2 gene mutations. In contrast, the IC50 of HG106 was 6.88 μM in KYSE150 cells and 8.79 μM in KYSE450 cells, which had a wild-type NRF2 genetic background. Overall, these results indicated that G6PD and SLC7A11 could be potential therapeutic targets in ESCC cells with an abnormal CKN axis.

**Figure 3.**
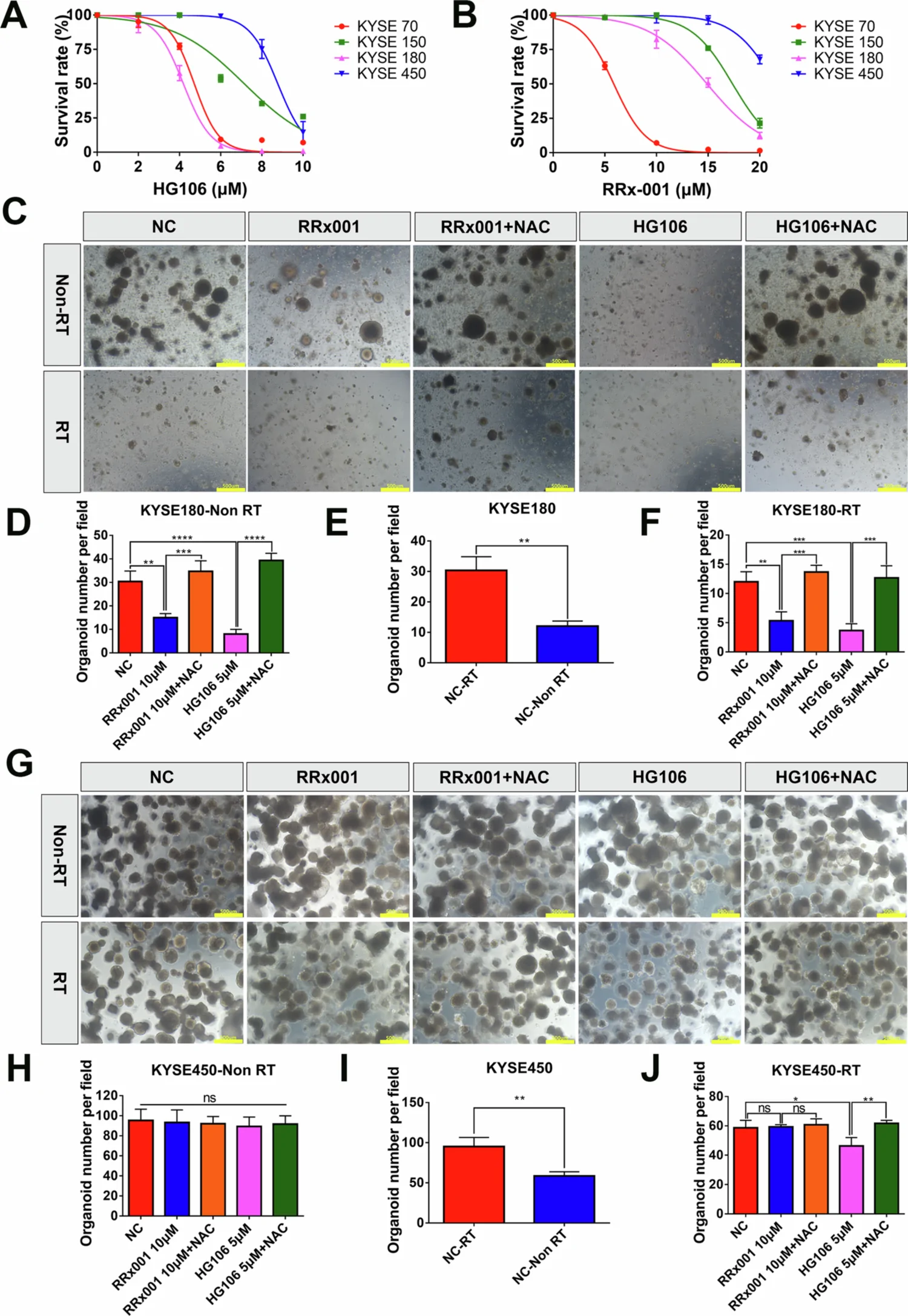
Sensitivity of ESCC cell lines and corresponding organoids with different NRF2 status to G6PD and SLC7A11 inhibitors. (A, B) Sensitivity of KYSE 70, KYSE 180, KYSE 150, and KYSE 450 to G6PD inhibitor RRx-001 and SLC7A11 inhibitor HG106. (C–J) Radiosensitivity of organoids derived from KYSE 180 and KYSE 450 under treatment of RRx-001 at 10  μM and HG106 at 5 μM, and the rescue effect of the antioxidant NAC at 1 mM. Figure C and G are representative graphs of organoids derived from KYSE 180 and KYSE 450, respectively. Scale bar 500 μm. *p < 0.05, **p < 0.01, ***p < 0.001, ns non-significant. NAC, *N*-Acety-L-Cysteine.

Next, we examined the impact of RRx-001 or HG-106 on ESCC organoids with either a wild-type or an aberrant CKN axis. The ex vivo ESCC organoid system used in our laboratory represented the composition and structure of in vivo ESCC tissue.^41^ ^,^ ^48^ We treated KYSE180 (NRF2mut) and KYSE450 (NRF2wt) organoids with 10 μM of RRx001 or 5 μM of HG106 (for details, see Materials and Methods). As shown in Figure 3C–D and 3G–H, treatment with either compound inhibited the organoid formation and reduced the size of the KYSE180 (NRF2mut) organoids more than the KYSE450 (NRF2wt) organoids. These effects on the KYSE180 (NRF2mut) organoids could be reversed by co-treating them with *N*-acetylcysteine (NAC), a potent antioxidant that exerts intercellular oxidation. Thus, consistent with the results from two-dimensional cultured cells, RRx-001 inhibited the key enzyme G6PD in the pentose phosphate pathway (PPP), and HG-106 inhibited the key enzyme SLC7A11 in glutathione (GSH) anabolism in ESCC organoids harboring an aberrant CKN axis, which not only abolished cell proliferation but also caused cell death. Taken together, these results indicated that G6PD and SLC7A11 could serve as pro-oxidative therapeutic targets in ESCC.

Previous studies demonstrated that DNA damage treatment strategies, such as radiotherapy (RT) or Adriamycin, could be effective treatment strategies for many cancers, including ESCC.^49^ ^,^ ^50^ RT induced ROS, which led to pro-oxidative effects in cells, such as DNA damage, cell cycle arrest, and cell death.^49^ These effects were mechanistically similar to those observed with G6PD or SLC7A11 inhibition in ESCC cells. Therefore, we hypothesized that RT could have a synergistic effect with G6PD and SLC7A11 inhibition, particularly in ESCC cells with an aberrant CKN axis. To test this hypothesis, we treated KYSE 180 and KYSE 450 organoids with RRx-001 or HG106 in combination with RT. As shown in Figure 3C, 3E, 3G, and 3I, RT treatment alone significantly inhibited the growth of both KYSE 180 and KYSE 450 organoids. Combination treatment with RT and either RRx-001 or HG106 nearly eliminated all KYSE 180 organoids with an aberrant CKN signaling pathway. However, the addition of NAC significantly reversed the effects of RT-RRx001- and RT-HG106-dependent killing effects on the organoids (Figure 3C, 3F). In contrast, no additional killing effects were observed in KYSE450 organoids with a wild-type CKN axis that were treated with RRx-001 in combination with RT (Figure 3G, 3J). Additional killing effects were observed in KYSE 450 organoids treated with HG106 in combination with RT, though to a much lesser extent. These effects could also be reversed by the addition of NAC. To further demonstrate this result, we used Trolox, another widely used antioxidant, to treat the organoids, and found a comparable rescue effect (Figure S3F–G). Taken together, these results indicated that G6PD and/or SLC7A11 inhibitors could be optimized as sensitizers for RT in combination therapy with CKN axis-aberrant ESCCs.

### A p53DN mutant, p53^R246W^, induces oxidation tolerance in D3 cell, independent of the CKN axis

TP53 mutations were highly prevalent in ESCC, and mutant p53 had pleiotropic effects on the regulation of redox balance. However, the precise functions and mechanisms by which p53 was involved in regulating redox balance and homeostasis, particularly its relationship with the CKN axis, remain unclear. To investigate whether there existed interplays between the CKN axis and p53 in the regulation of redox balance, we analyzed the TCGA ESCC genomic database. We found that 75.4% (249/317) of ESCC patients harbored a p53 mutation. Among these patients, 62.7% (156/249) had missense mutations concentrated in the DNA-binding domain (DBD) (Figure S4A–B). To determine the role of p53 mutations in regulating the redox balance and homeostasis in cells with a clear genetic background, we generated a D3 cell line that expressed a FALG-tagged p53DN mutant (p53^R246W^), a mutation commonly detected in human cancers, including ESCC (Figure S4C).^51^ Immunoblotting and immunofluorescence using the M2 antibody revealed the accumulation of the FALG-tagged p53^R246W^ mutant protein in the nuclei of the D3-p53^R246W^ cells (Figure 4A and 4B). Then, we treated the D3-p53^R246W^ cells with 0.2 μM Adriamycin and examined the cellular responses induced by the treatment. Immunoblotting analysis showed that Adriamycin caused *γ*-H_2_AX accumulation in both D3 parental cells and D3-p53^R246W^ cells. However, Adriamycin did not affect the levels of *p*-CHK1, p21, or pH3 in the D3-p53^R246W^ cells compared to the D3 parental control cells (Figure S4D). These results indicated that Adriamycin treatment was unable to initiate the DNA damage response process in D3-p53^R246W^ cells. The p53^R246W^ mutant suppressed the physiological function of the wild-type p53 protein, reflecting the p53 DN effect. Next, we examined how the p53^R246W^ mutant affected cell proliferation. Growth curves revealed that D3-p53^R246W^ cells exhibited accelerated growth compared to parental D3 cells (Figure S4E). Immunoblotting revealed significantly higher levels of ERK1/2 phosphorylation in D3-p53^R246W^ cells (Figure S4F). These results supported that the p53^R246W^ mutant enhanced the proliferation ability of the D3 cell, displaying a gain-of-function (GOF) effect in D3-p53^R246W^ cells. However, flow cytometry analysis indicated that the p53^R246W^ mutant had no impact on the cell cycle profiles of D3 cells, indicating that the p53^R246W^ mutant did not affect a specific cell cycle phase when expressed in D3 cells (Figure S4G–H). Taken together, these results, along with the high p53 mutation rate in ESCC, demonstrated that p53^R246W^ had a DN effect on p53wt, ultimately displaying GOF effect in D3 cells.

**Figure 4.**
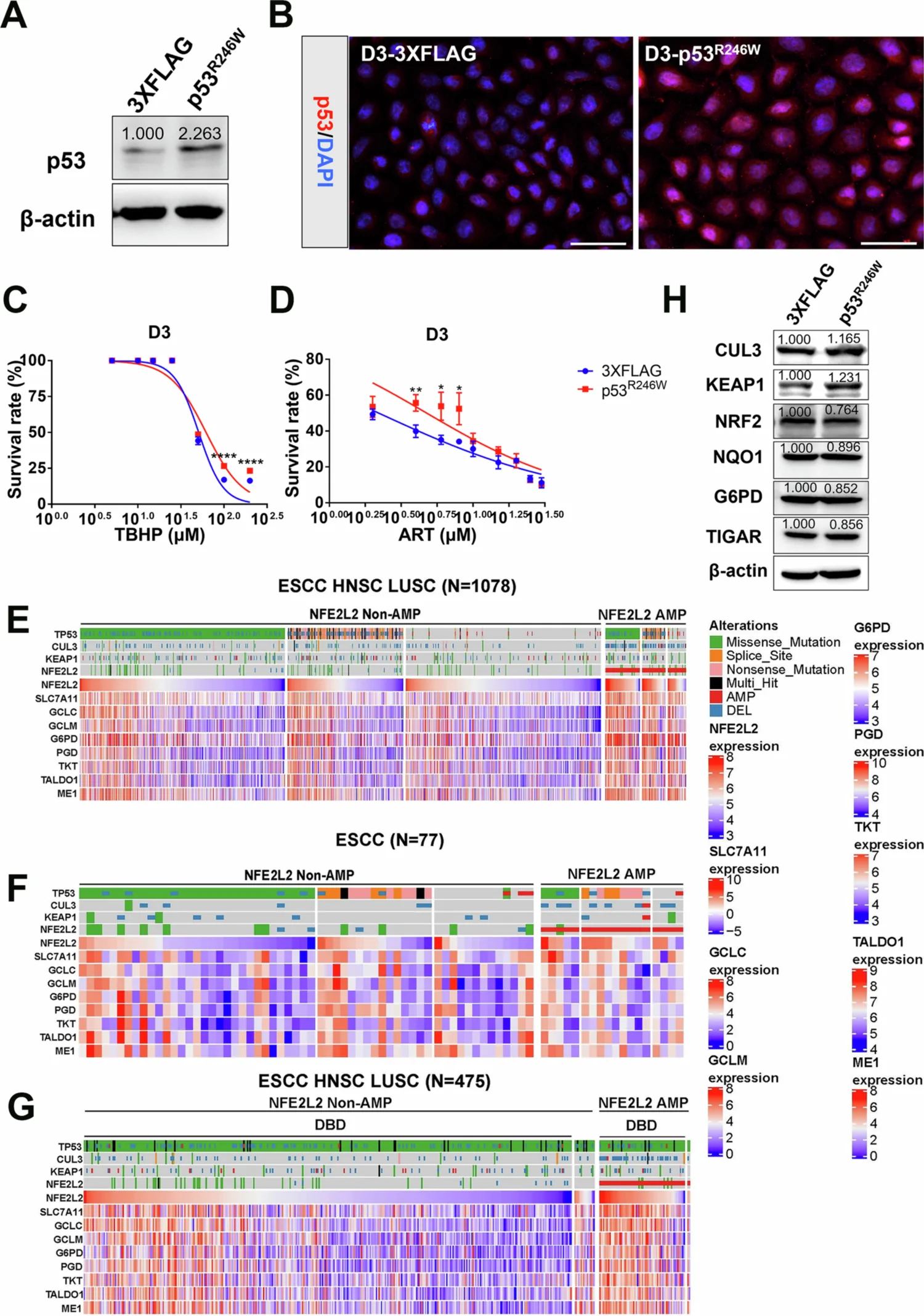
The p53^R246W^ mutant introduced oxidation tolerance in D3 cell independent of NRF2. (A) Immunoblots of total p53 protein accumulation after p53^R246W^ mutant transfection in D3 cell. (B) Immunofluorescence staining of p53 nuclei localization after p53^R246W^ mutant transfection in D3 cell. Scale bar 30 μm. (C, D) Cell viability changes of D3 cell to TBHP (C) and ART (D) at gradient concentration after p53^R246W^ transfection. (E–G) Bioinformatical analysis of associations between TP53 and NFE2L2 alterations and associations of relevant NRF2 downstream genes from the TCGA database. All of the samples were divided into NFE2L2 non-amplification and NFE2L2 amplification groups for the first step, then TP53 was divided into missense mutation group, splice mutation and multi hit group, and nonsense mutation group, followed by corresponding variations of other relevant genes. (H) Immunoblots of CUL3-KEAP1-NRF2 axis and relevant proteins after p53^R246W^ mutant transfection in D3 cell. *p < 0.05, **p < 0.01, ***p < 0.001. ART, artesunate. AMP, amplification. DEL, deletion. DBD, DNA-binding domain. ESCC, esophageal squamous cell carcinoma. HNSCC, head and neck squamous cell carcinoma. LUSC, lung squamous cell carcinoma.

To determine the anti-oxidative capacities of D3-p53^R246W^ cells and their parental control D3 cells, we treated these cells with exogenous oxidants. As shown in Figure 4C–D, the IC50 values for TBHP and ART were significantly higher in the D3-p53^R246W^ cells than in the D3 cells. These results indicated that the expression of the p53^R246W^ mutant significantly enhanced oxidation tolerance in D3 cells. Next, we explored whether this effect depended on the CKN axis and whether the two pathways interacted to control the redox balance of D3 cells. First, we performed bioinformatic analysis of mRNA expression and genetic alteration data from TCGA ESCC and SCC samples. We found no correlation between the p53 pathway and the CKN axis, in terms of either mRNA expression or genetic alterations (Figure 4E–G). Immunoblotting analysis showed no significant changes in the expression of CUL3, KEAP1, NRF2, NQO1, and G6PD in D3-p53^R246W^ cells compared to the parental control D3 cells (Figure 4H). The expression levels of the NRF2 ubiquitination regulatory factors CUL3 and KEAP1 remained unchanged, and there was no evident intracellular accumulation of NRF2 protein. Consistently, the canonical NRF2 downstream target NQO1 also exhibited no significant upregulation. To confirm these results further, we performed RT-qPCR for the canonical NRF2 downstream genes Gclm, Sod1, Pgd, Tkt, and Taldo1 and obtained similar findings (Figure S3E). While some genes exhibited significant expression differences between D3 and D3-p53^R246W^ cells, the fold changes were generally no greater than two, which was below the threshold typically considered biologically meaningful for NRF2-dependent transcriptional activation.^52^ These results indicated that, although D3-p53^R246W^ cells exhibited high oxidation tolerance when treated with exogenous oxidants, the CKN axis was not dysregulated in these cells. In addition, it was of interest that the expression of TIGAR, a previously reported downstream target of p53 involved in maintaining the redox balance, was also not perturbed in D3-p53^R246W^ cells, suggesting the existence of an alternative target(s) and/or pathway(s) involved in the high oxidation tolerance of the D3-p53^R246W^ cells (Figure 4H).^53^

### The EGFR-ERK-ETS1-SLC7A11 (EEES) axis mediated GSH anabolism contributed to the high oxidation tolerance of the D3-p53^R246W^ cells

To identify alternative targets and/or pathways involved in the high oxidative tolerance of D3-p53^R246W^ cells, we investigated whether GSH metabolism was altered in these cells. RT-qPCR and immunoblotting analyses revealed that SLC7A11 was markedly upregulated in D3-p53^R246W^ cells compared to the parental D3 control cells (Figure 5A–B). These results suggested that this p53 mutant may facilitate SLC7A11 expression at the transcriptional level, thereby enhancing GSH anabolism and contributing to the tolerance of cellular oxidation in D3 cells. Consistently, the GSH quantification assay revealed increased GSH anabolism in D3-p53^R246W^ cells compared to the parental control D3 cells (Figure 5C). Additionally, an RNAi assay showed that SLC7A11 knockdown significantly decreased tolerance to TBHP in D3-p53^R246W^ cells (Figure 5D–E). Based on these findings, we concluded that the expression of p53^R246W^ enhanced SLC7A11 expression, thereby altering GSH anabolism and increasing antioxidant capacity in D3 cells. Previous studies have indicated that, in addition to NRF2, ETS1 was an important transcription factor for SLC7A11 during the oncogenic anti-oxidation process.^54^ ETS1 transcriptional activity was activated by the phosphorylation of ETS1 by the EGFR-ERK cascade, followed by its nuclear translocation.^54^ As many of the components of the EGFR-ERK signaling cascade were transcriptionally controlled by p53, especially its dominant-negative mutants (e.g. p53^R246W^) during the oncogenic antioxidant process, we hypothesized that the expression of p53^R246W^ activated the EGFR-ERK cascade, resulting in the phosphorylation of ETS1 followed by its nuclear translocation, ultimately enhancing SLC7A11 expression and GSH anabolism and increasing antioxidant activity in D3 cells. We tested this hypothesis and showed that the expression of EGFR, phosphorylation of EGFR, phosphorylation of ERK, and phosphorylation of ETS1 at Thr38 were significantly increased in p53^R246W^ cells compared to the parental control D3 cells (Figure S4F, Figure 5A). To further confirm this hypothesis, we treated D3 cells with the ERK activator Sorafenib and found significant upregulation of ETS1 phosphorylation and SLC7A11 expression (Figure 5F). Consistently, the ERK inhibitor Trametinib inhibited the phosphorylation of ERK1/2 and ETS1, subsequently decreasing the expression of SLC7A11 (Figure 5G). Moreover, knocking down ETS1 expression using RNAi significantly decreased SLC7A11 expression and anti-oxidative capacity of D3-p53^R246W^ cells (Figure 5H-I). Taken together, these results demonstrated that the expression of p53^R246W^ activated the EGFR-ERK cascade, resulting in the phosphorylation of ETS1 followed by its nuclear translocation, ultimately enhancing SLC7A11 expression and GSH anabolism and increasing antioxidant activity in D3 cells.

**Figure 5.**
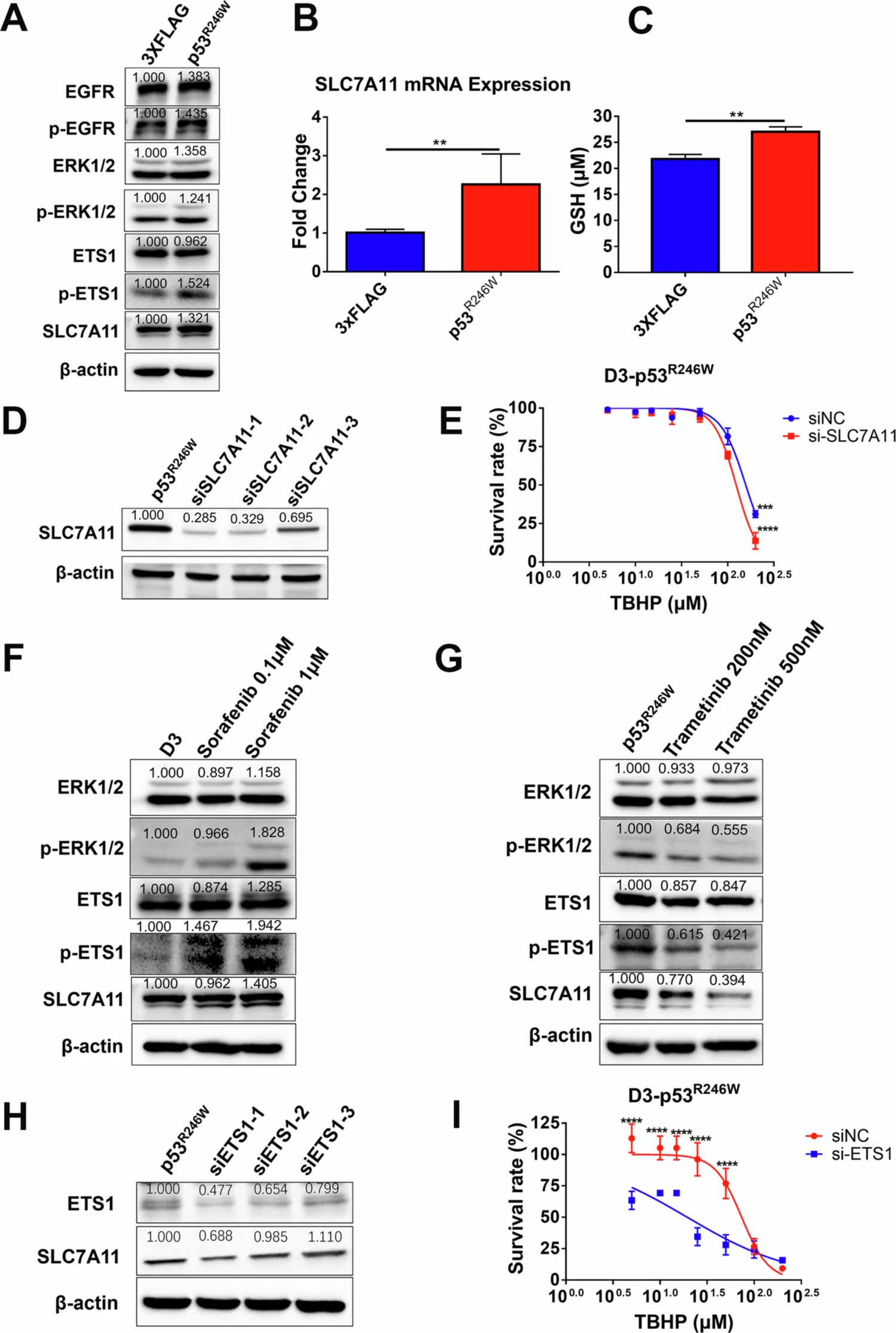
EGFR-ERK-ETS1-SLC7A11 axis-mediated GSH anabolism contributed to the antioxidative phenotype of D3-p53^R246W^ cell. (A) Immunoblots of EGFR-ERK1/2-ETS1-SLC7A11 axis change after p53^R246W^ mutant transfection in D3 cell. (B) RT-qPCR analysis of Slc7a11 mRNA expression change after p53^R246W^ mutant transfection in D3 cell. (C) GSH anabolism changes after p53^R246W^ mutant transfection in D3 cell. (D) Knockdown efficacy of Slc7a11 using RNAi at the protein level in p53^R246W^-D3 cell. Based on the result, siSLC7A11-1 was selected for further experiment. (E) Sensitivity variation of p53^R246W^-D3 cell to TBHP after Slc7a11 knockdown using RNAi. (F, G) Immunoblots of ETS1 and SLC7A11 change after treatment with *p*-ERK1/2 activator Sorafenib and *p*-ERK1/2 inhibitor Trametinib. (H) Knockdown efficacy of Ets1 using RNAi at protein level and the corresponding change tendency of SLC7A11 in p53^R246W^-D3 cell. Based on the result, siETS1-1 was selected for further experiment. (I) Sensitivity variation of p53^R246W^-D3 cell to TBHP after Ets1 knockdown using RNAi. **p < 0.01, ***p < 0.001, ****p < 0.0001.

### The effects of the oxidation inducer ART on the balance of oxidants in vivo and chemoprevention in the NMBzA-induced rat ESCC model

As the two major redox balance regulators, the CKN axis and p53, were found to control the redox balance by regulating the PPP and GSH anabolism independently in esophageal keratinocytes in vitro, we tested whether manipulating the redox balance could impact the oncogenic antioxidant process effectively in vivo. To this end, we employed ART in the NMBzA-induced rat ESCC model developed in our laboratory to evaluate the impact of the oxidation inducer on the balance of oxidants and chemoprevention in vivo.^48^ ^,^ ^55^ ^,^ ^56^ The detailed experimental procedure was shown in Figure S6A, where 32 Fisher 344 male rats were randomly divided into four groups, group A to group D. Rats in group A were administered with ART for 35 weeks only. Rats in group B were administered with NMBzA for 8 weeks only. Rats in group C were administered with ART for 2 weeks, followed by NMBzA for 8 weeks together, and then ART for 37 weeks. Rats in group D were administered with ART and NMBzA together for 8 weeks, followed by ART for 37 weeks. ART was well tolerated throughout the experiment, as evaluated by monitoring the body weight of rats (Figure S6C). All animals were sacrificed at week 27 and week 37 after NMBzA was administered to them and animal esophageal tissues were collected and analyzed by gross photography, hematoxylin and eosin (H&E) staining, or immunohistochemistry.

Gross pathological examination of the samples taken from the rat esophagi showed that treatment with ART alone did not cause any tumors at weeks 27 and 37. In contrast, treatment with NMBzA alone caused rough lesions in the rats' esophagi at week 27, which then developed into tumors at week 37. However, the rats treated with ART before or with NMBzA together had fewer rough lesions and tumors than rats treated with NMBzA only. Histological H&E analysis showed that these lesions and tumors displayed the dynamic transitions from normal esophageal epithelia to hyperplasia (excessive cell growth), dysplasia (abnormal cell growth), papilloma (small growth on the surface of the tissue), and squamous cell carcinomas (cancer that starts in the squamous cells of the esophagi) (Figure S6B, Figure 6A–I).

**Figure 6.**
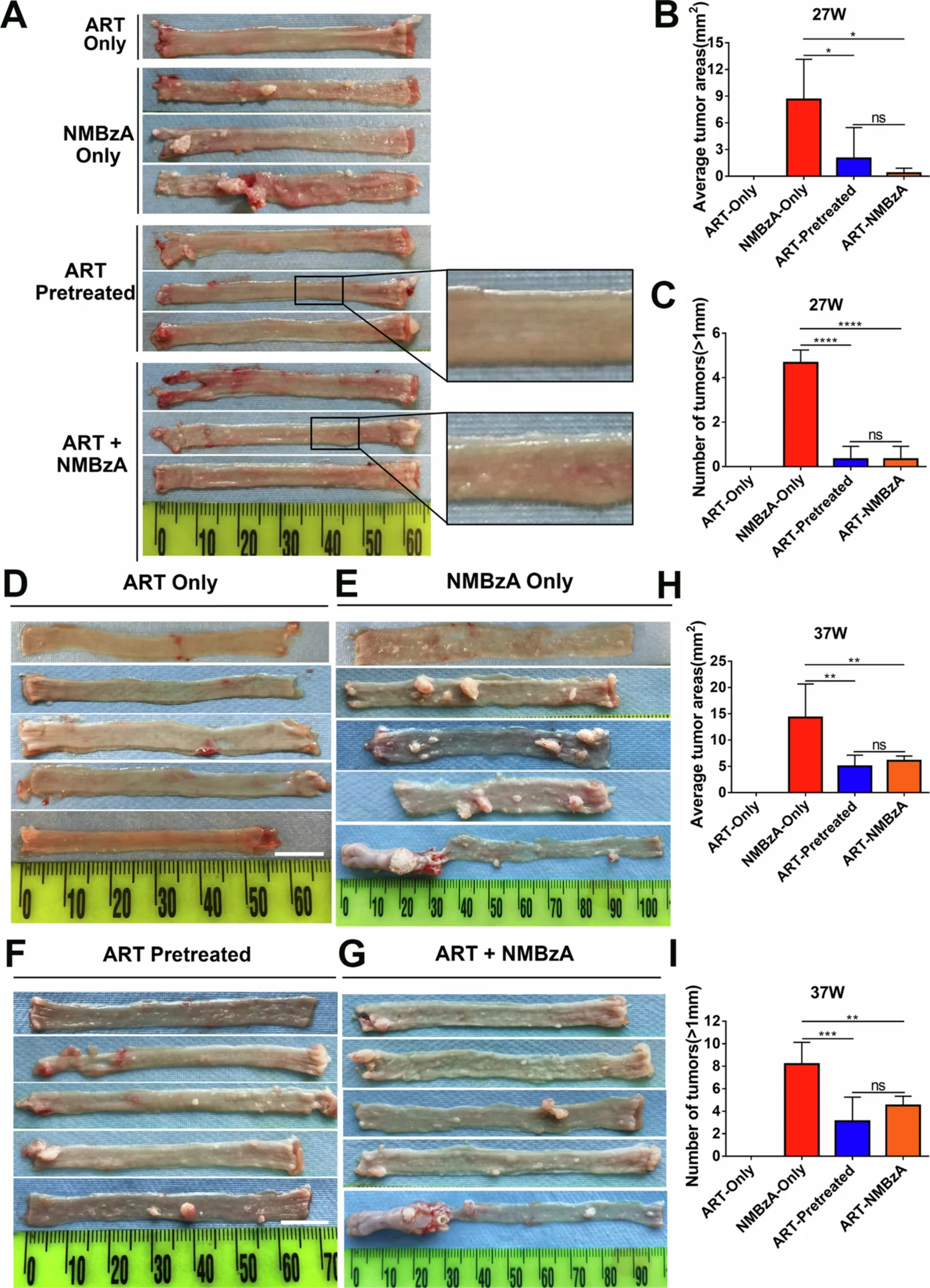
Chemoprevention effect of ART in the NMBzA-induced rat ESCC model. (A–C) The gross rat esophagi pathological specimen photograph and quantification of tumorous variations after NMBzA and/or ART treatment at 27th week. (D-I) The above changes at 37th week in different groups. *p < 0.05, **p < 0.01, ***p < 0.001, ****p < 0.0001. ART, artesunate.

We performed immunohistochemistry (IHC) using several anti-oxidative markers to determine the effects of ART in the tissues of the NMBzA-induced rat ESCC model. We found that the proportion of NRF2 nuclear-positive cells was significantly higher in the NMBzA-alone treated group than in the ART-treated or ART- and NMBzA-treated groups, indicating that ART plays a crucial role in the process of ESCC carcinogenesis by activation of NRF2 (Figure S6D–E). In contrast, p53 nuclear-positive cells were rarely observed in samples from any group. However, the intensity of p53 staining was higher in the NMBzA-treated group than in the ART only or ART-and NMBzA-treated groups (Figure S6E). These results indicated that the CKN axis plays a major role, whereas p53 plays a minor role in regulating the redox balance of the rat ESCC carcinogenesis model induced by NMBzA. Thus, both G6PD and SLC7A11-positive staining intensities were higher in the NMBzA-treated group than in the ART-only and ART-and-NMBzA-treated groups (Figure S6E). Taken together, these results revealed that balancing oxidant levels via the CKN axis with oxidant inducers in vivo could contribute to chemoprevention in the NMBzA-induced rat ESCC model.

## Discussion

Accumulating evidence has demonstrated that the regulation of redox balance and homeostasis plays a critical role in human tumorigenesis, including ESCC carcinogenesis.^24^ ^,^ ^33^ ^,^ ^57^ Previously, we and others have shown that normal esophageal keratinocytes generally develop robust mechanisms to combat oxidative damage and/or lesions.^19^ ^,^ ^24^ In contrast, ESCC cells have evolved reprogrammed antioxidant systems to adapt to their enriched oxidant environment through genetic alterations and epigenetic changes. Two critical regulators of redox balance and homeostasis, the CKN axis and the p53 pathway, have been shown to reprogram oxidative metabolism via abnormal NRF2 activation and/or p53DN effects caused by genetic alterations of the CKN axis and the p53 gene, which allow ESCC cells to avoid lethal oxidative lesions.^11^ ^,^ ^21^ ^,^ ^57^ However, the precise roles of the CKN axis and the p53, especially their interplay, in these processes remain controversial and elusive.^21^ ^,^ ^26^ ^,^ ^58-60^ In this study, we confirmed that frequent genetic alterations of the CKN axis and p53 gene mutations, which lead to NRF2 transcriptional activation and p53DN function, are common events in ESCC. We investigated how the activation of NRF2 and/or the expression of the p53DN mutant p53^R246W^ alone or in co-operation affects the redox balance. We examined whether the CKN axis and the p53 pathway interplay with each other to maintain redox homeostasis in esophageal cells. Our results reveal that activating NRF2 with KI696, a high-affinity small molecule that inhibits KEAP1-NRF2 interaction, together with the oxidizing agents TBHP or ART, increases the antioxidant capacity in esophageal cells.

Our RNA-seq analysis demonstrated that detoxification and metabolism in esophageal D3 keratinocytes treated with KI696 resulted in NRF2 nuclear translocation and transcriptional activation, which in turn increased the gene expression of the NRF2 downstream targets involved in PPP and GSH anabolism, two critical cellular antioxidant defense mechanisms. Previous studies have shown that the PPP and GSH anabolism are predominately enhanced biological processes after NRF2 activation, and that they are functionally indispensable. Since the detoxification components, such as Thioredoxin and Heme Oxygenase 1, are confirmed antioxidant factors that play end-effect roles in cell detoxification, we did not incorporate them into our study.^45^ Instead, we focused on the PPP and GSH anabolism due to their antioxidant processes of synthetically producing NADPH and GSH.^28^ ^,^ ^29^ We found that the critical components G6PD in the PPP and SLC7A11 in the GSH anabolism synergize with the oxidizing agents TBHP and ART and the DNA-damaging treatment radiotherapy to kill esophageal cells, particularly ESCC cells with CKN mutations. Consistent with our findings, metabolomics studies by Kitano et al.^22^ and Mitsuishi Y et al.^23^demonstrated that inducing NRF2 activation increased the expression of G6PD and SLC7A11 in mouse esophageal epithelial cells and ESCC patient samples. Taken together, these results suggest that G6PD and SLC7A11 could be therapeutic targets for NRF2-activated ESCC patients, whether due to induction via oxidizing/DNA-damaging agents or genetic alterations in the CKN axis.

Previous studies have revealed that p53DN mutants have a complex relationship with NRF2 that involves regulating redox balance.^61^ However, many studies contradict each other. To further investigate whether p53DN mutants co-regulate and/or interplay with NRF2, we expressed a p53DN mutant, p53R246W, in D3 cell. It has been proven that p53DN mutants not only disrupt the function of wild-type p53, the DN effect, but also augment its intrinsic ability, the GOF effect, as demonstrated by the impaired DNA damage response signaling and enhanced cell proliferation in our results. Importantly, we discovered that p53^R246W^ expression in D3 cells increases the anti-oxidative effect by upregulating SLC7A11, independent of NRF2. Our results differ from those of some other studies.^26^ ^,^ ^62^ The reason for these discrepancies is currently unknown. One possible reason is the use of different p53DN mutants, as not all p53 mutants have equivalent functions.^63^ Liu et al. showed that accumulated mutant p53 proteins bound to NRF2, mechanically suppressing SLC7A11 transcription and GSH synthesis by NRF2 and enhancing tumor sensitivity to oxidative stress.^26^ Su et al. suggested that another hotspot p53 mutant, R175H, significantly upregulates SLC7A11 expression by interacting with BACH1 to alleviate the repressive effect on gene transcription at the chromatin level.^62^ These protein–protein interactions and chromatin competition are important under certain conditions. Furthermore, since both NRF2 and p53 are thiol-based sensors, oxidation and cellular stress may substantially influence the present results and should not be neglected.^10^ ^,^ ^11^ Nevertheless, our results indicated that p53^R246W^ expression in D3 cells resulted in SLC7A11 upregulation via the EEES axis, thereby increasing the antioxidant effect. Since p53^R246W^ was previously reported to induce high expression of miR-27a and miR-155, which increased EGFR expression, one might expect a link between p53^R246W^ expression and SLC7A11 upregulation via the EEES axis to be mediated by these microRNA (miRNA) molecules. Thus, SLC7A11 could also be considered as a therapeutic target for p53-mutated ESCCs. However, the specific regulation of p53^R246W^ on EGFR transcription, as well as evidence of direct ETS1 binding to the SLC7A11 promoter in D3-p53^R246W^ cell is lacking. While ChIP-qPCR validation for ETS1 binding to the SLC7A11 promoter was not conducted in the present study, bioinformatics analysis reliably predicted the presence of bona fide ETS1 binding sites in this promoter region (Figure S5).^64^ ^,^ ^65^ Future studies must elucidate the precise molecular mechanism by which the expression of p53^R246W^ in D3 esophageal keratinocytes results in an antioxidant effect through the upregulation of SLC7A11.

We employed an exogenous oxidant, ART, in an NMBzA-induced rat ESCC model to learn more about the role of redox balance regulation in ESCC carcinogenesis. ART is an anti-malarial agent with a peroxide bridge that can be activated by intracellular Fe^2+^ to produce ROS.^43^ Interestingly, our in vitro study revealed that ART functions as an oxidant inducer, reducing cell variability upon NRF2 activation in D3 cells. However, ART application in the NMBzA-induced rat ESCC model clearly inhibited esophageal tumor initiation and progression in vivo. One possible explanation for our results comes from a recent study demonstrating the selective elimination of NRF2-activated cells through competition with neighboring cells in the esophageal epithelium of conditional KEAP1 knockout mice.^24^ The authors of this study showed that cells with high NRF2 activation and cells with lower NRF2 nuclear expression coexisted in the esophageal epithelium. NRF2-activated cells exhibited weaker attachment to the basement membrane and were gradually eliminated from the epithelium. In contrast, neighboring cells with low NRF2 activation exhibited accelerated proliferation and began to dominate the epithelium, but they accumulated DNA damage that triggered carcinogenesis upon exposure to carcinogens. Given that ART is a strong oxidant inducer, our results could have similar effects.

Recently, we demonstrated that NRF2 activation occurs gradually during the initiation and progression of esophageal carcinogenesis from normal tissue to hyperplasia, dysplasia, and finally, carcinoma, in vivo.^62^ This finding is consistent with genomic sequencing data showing frequent NRF2 pathway activation in ESCC.^16-18^ Together with the results presented in this study, these findings suggest that NRF2 may have dual functions during tumor evolution. Future studies should focus on the exact causal role of NRF2 at different stages of tumor development. It is conceivable that ART treatment may increase the level of intracellular oxidative stress and activate NRF2 in the NMBzA-induced ESCC carcinogenesis model. Along with increasing Col17A1 expression, NRF2-activated cells are eliminated from the esophageal epithelium, exhibiting chemopreventive properties against tumor initiation and progression.^24^ ^,^ ^66^ Thus, the chemopreventive effect of ART may be due to its oxidizing effect rather than its antioxidant effect. Consistently, ART has previously been tested for chemoprevention in different carcinogen-induced animal models, including those of liver, colorectal, and breast cancers, and has shown a consistent preventive effect.^63^ ^,^ ^66^ ^,^ ^67^ However, it remains unclear whether ART exerts its chemopreventive function via selective elimination at the tissue level or direct tumor cell killing. In this study, we observed that the chemopreventive effect of ART appeared to be more pronounced during the first 27 weeks (tumor initiation stage) than during the subsequent 28–37 weeks (tumor progression stage) (Figure 6). As time passed and the process advanced to the late stage of carcinogenesis, the effect of ART was reduced along with tumor growth. Therefore, we speculate that ART could serve a chemopreventive role by eliminating NRF2-activated cells during the first 27 weeks and a direct killing role by disrupting the redox balance of vulnerable tumor cells during the subsequent weeks.

In summary, this study aims to systematically elucidate the key redox regulators involved in ESCC. By integrating classic signaling pathways, including the NRF2, p53, and EGFR/ERK pathways, as well as the biological function of SLC7A11, we clarified the regulatory relationships among these molecules. Under the tested conditions, we found no evidence of direct regulation between NRF2 and the p53^R246W^ mutant, which modulates redox balance/homeostasis during ESCC tumorigenesis. NRF2 and p53^R246W^ both exert regulatory effects through an SLC7A11-dependent metabolic mechanism. Furthermore, the EGFR-ERK-ETS1-SLC7A11 signaling axis primarily mediates the antioxidant function of the p53^R246W^ mutant. Given the fact that both NRF2 and p53 are critically involved in ESCC carcinogenesis, targeting cellular redox homeostasis is a promising therapeutic strategy for preventing and treating the disease.

## Materials and methods

### Cell culture and organoid formation

The immortalized D3 rat esophageal epithelial cell was established and maintained by our laboratory. D3 cell was cultured in DMEM/F12 (3:1) medium containing 10% fetal bovine serum. Detailed medium components were described as before.^41^ ^,^ ^68^ The ESCC cell lines (KYSE70, KYSE180, KYSE150, and KYSE450) were given by Dr. Y Shimada of Kyoto University as gifts and cultured in RPMI-1640 medium containing 10% fetal bovine serum. All the ESCC cell lines were authenticated by short tandem repeats profiling. The D3 cell line had been sequenced before and confirmed to be rat-originated.^41^ All the used cell lines were affirmed to be mycoplasma-free and cultured at 37 °C with 5% CO_2_. HG-106 (synthesized by Lidekang Pharmaceutical Technology Co., LTD),^46^ KI696 (MedChemExpress, HY-101140), Artesunate (Macklin, MFCD00866204), RRx-001 (Selleck, S8405), Adrimycin (Selleck, S1208), Sorafenib (Selleck, S7397), and Trametinib (Selleck, S2673) were dissolved in DMSO (Sigma-Aldrich, C6164) and used at specific final concentrations. tBHP (Sigma-Aldrich, 458139) was diluted from the mother liquor each time it was used. Organoid culture was described as before,^68^ we removed the Rock inhibitor (Y27632) from the medium and supplemented IGF-1 (PEPROTECH, 100-11), FGF-10 (PEPROTECH, 100-26), Bovine Pituitary Extract (Gibico, 13028014), Gastrin (R&D Systems, 3006/1) for ESCC cell line-derived organoids.

Radiotherapy for ESCC cell line-derived organoids was performed using a Varian Truebeam linear accelerator by 6 MeV X-ray at the source to surface distance of 100 cm. All the organoids accepted radiotherapy at day 8–10 once daily, for 3 Gy each time in combination with/without the administration of HG-106 at 5 μM or RRx-01 at 10  μM and *N*-acetylcysteine at 1 mM or Trolox at 100 μM. Photographs were taken at day 20.

Lipofectamine RNAiMAX (Invitrogen, 13778150) was used for siRNA transfections, and siRNA sequences were as follows:

(1) G6pd

siRNA-1: 5'-CCAAGCUAGAGGAGUUCUUTT-3'

siRNA-2: 5'-CCAUCUGGAAUCGAGACAATT-3'

siRNA-3: 5'-GGUCUACCACUGCUACCUUTT-3'

(2) Slc7a11

siRNA-1: 5'-CCAGAUAUGCAUCGUCCUUTT-3'

siRNA-2: 5'-GCUCACAGCAAUUCUGAUATT-3'

siRNA-3: 5'-CAUGAACGGUGGUGUGUUUTT-3'

(3) Ets1

siRNA-1: 5'-GGUGAUGUGGGCUGUGAAUTT-3'

siRNA-2: 5'-CCAGAAGUUCUGUAUGAAUTT-3'

siRNA-3: 5'-GCUUCAUCACAGAGUCCUATT-3'

Rat p53^R246W^ mutant was constructed using pCDH-CMV-Trp53 (rat, R246W)-3Flag-EF1-hygro vector (Figure S4C), referring to NM_030989 sequence. The specific sequence was uploaded as a supplement. The lentivirus was used for the introduction of the plasmid using polybrene at an MOI of 50.

### Cell viability assay and cell cycle analysis

Cell Counting Kit-8 (Dojindo, Kumamoto, CK04) was used for cell determination, and the absorbance at 450 nm was measured by the Multiskan GO Spectrophotometer (Thermo Fisher Scientific, Karlsruhe). Cell cycle analysis was performed using FACS (fluorescence-activated Cell Sorting) after the cells were dyed with PI (propidium iodide, 1:1000 diluted by PBS). FlowJo software was used for cell cycle analysis.

### ROS, NADPH, and GSH detection

ROS production was detected using the ROS Assay Kit (Beyotime, S0033S) following the manufacturer’s instructions. After incubating with reagents, DCF fluorescence intensity was quantified by FACS. NADPH and GSH detection assays were operated using NADP/NADPH Assay Kit (Dojindo, Kumamoto, N510) and GSSG/GSH Quantification Kit II (Dojindo, Kumamoto, G263) following the manufacturer’s instructions. 5-SSA (5-Sulfosalicylic acid dihydrate, Dojindo, Kumamoto, A610610-0050) was used at 0.5%. For NADP/NADPH detection, the absorbance was measured at 450 nm, and the wavelength for GSSG/GSH determination was 415 nm.

### Immunoblotting, quantitative PCR, and immunofluorescence

The procedures for immunoblotting, quantitative PCR (q-PCR), and immunofluorescence (IF) assays were described previously.^68^ For immunoblotting, the cells were lysed using RIPA (Beyotime, P0013B) on ice and centrifuged at 12,000 rpm for protein extraction. A phosphatase inhibitor cocktail (Beyotime, P1081) was supplemented when necessary. Primary antibodies used were as following: NRF2 (Abcam, ab62352, 1:1000), NQO1 (Proteintech, 67240-1-Ig, 1:10,000), G6PD (Abcam, ab210702, 1:1000), SLC7A11 (Invitrogen, PA1-16893, 1:800), p53 (Proteintech, 60283-2-Ig, 1:5000), FLAG (Sigma–Aldrich, F3165, 1:1000), CUL3 (Proteintech, 11107-1-AP, 1:1000), KEAP1 (Proteintech, 10503-2-AP, 1:2000), TIGAR (SANTA CRUZ, sc-166290, 1:1000), EGFR (Abcam, ab52894, 1:2000), Phospho-EGFR-Y1068 (Abcam, ab32430, 1:1000), ERK1/2 (Beyotime, AF1051, 1:1000), Phospho-ERK1-Thr202/Tyr204+ ERK2-Thr185/Tyr187 (Beyotime, AF5818, 1:1000), ETS1 (CST, #14069, 1:1000), Phospho-ETS1-Thr38 (Sigma–Aldrich, SAB4503912, 1:500), MCM2 (abcam, ab108935, 1:2000), *γ*-H2Ax (CST, #9718, 1:1000), Phospho-ATM-Ser1981 (CST, #5883, 1:1000), Phospho-Chk1-Ser317 (CST, #12302, 1:1000), Phospho-Chk2-Thr68 (CST, #2197, 1:1000), *p*-H3 (CST, #53348, 1:1000), p21 (Abcam, ab109199, 1:1000), and *β*-actin (Proteintech, 66009-1-Ig, 1:10,000).

For q-PCR, mRNA reverse transcription was performed using the Prime-Script™ RT Reagent Kit (Takara, RR037A). Q-PCR was carried out using the TB Green Premix Ex Taq Taq™ (Takara, RR420A). GAPDH was used as a reference gene, and the primers used were as follows:

(1) Rat Nqo1

Forward: 5'-ACTCGGAGAACTTTCAGTACC-3'

Reverse: 5'-TTGGAGCAAAGTAGACTGGT-3'

(2) Rat G6pd

Forward: 5'-CTGACAACGTGGTCCTTGGC-3'

Reverse: 5'-GCTCATTCAGAGCTTTGCCAC-3'

(3) Rat Slc7a11

Forward: 5'-ATGTATGCATATGCTGGCTGG-3'

Reverse: 5'-TGTCAGGACATAGCCAACTGTG-3'

(4) Rat Gclm

Forward: 5'-CATCGTGAAAAGCAGAGGAATC-3'

Reverse: 5'-GGCCCCTGAGTTGTGAGC -3'

(5) Rat Sod1

Forward: 5'-AGCAGAAGGCAAGCGGTGAAC-3'

Reverse: 5'-CGTCCTTTCCAGCAGCCACATT-3'

(6) Rat Pgd

Forward: 5'-TCTGCGAGGCTTACCACTTGATGA-3'

Reverse: 5'-ATGAGTCCAGCTCTGTCTTGTTCCA-3'

(7) Rat Tkt

Forward: 5'-CTGAAGAGCAAGGACGACCAAGTG-3'

Reverse: 5'-TGCCTCGCCTATGCCACCTT-3'

(8) Rat Taldo1

Forward: 5'-CAAAGCAGCCCAGACCAGT-3'

Reverse: 5'-CACAGCCATTTGGTCCTCATT-3'

(9) Rat Gapdh

Forward: 5'-CATGCCGCCTGGAGAAAC-3'

Reverse: 5'-CCCAGGATGCCCTTTAGT-3'

(10) Homo G6PD

Forward: 5'-ACGATGACACTGCCTCCTACAAGC-3'

Reverse: 5'-GCTCTTTGAAGGTGAGAATGACGC-3'

(11) Homo SLC7A11

Forward: 5'-ATGCAGTGGCAGTGACCTTT-3'

Reverse: 5'-GGCAACAAAGATCGGAACTG-3'

(12) Homo NQO1

Forward: 5'-AACTGAAGGACCCTGCGAAC-3'

Reverse: 5'-AATGGCAGGGACTCCAAACC-3'

(13) Homo GAPDH

Forward: 5'-TGTTGCCATCAATGACCCCTT-3'

Reverse: 5'-CTCCACGACGTACTCAGCG-3'

For IF, cells were seeded on glass coverslips in a 24-well incubator. After fixation, the sections were incubated with primary antibodies and corresponding fluorescent secondary antibodies, then the sections were counterstained with DAPI and sealed with SlowFade Diamond Antifade Mountant (Thermo Fisher Scientific, P36982) for microscopic observation. The primary antibodies used were as follows: NRF2 (Abcam, ab62352, 1:200) and p53 (Proteintech, 60283-2-Ig, 1:800).

### Animal model establishment, hematoxylin & eosin staining, and immunohistochemistry

The Ethical Committee of the National Cancer Center/Cancer Hospital, Chinese Academy of Medical Sciences, approved the total animal experiments in this article (Approval Number: NCC2023A123). Fisher 344 (F344) rats were purchased from Vital River Laboratory Animal Technology (Beijing, China). Rats were 3–4 weeks old at the beginning of the study. Two rats were housed per cage, the temperature was 24 ± 2 °C, the relative humidity was 20, light and dark were cycled each for 12 h to keep a diel rhythm, standard rodent maintenance fodder (Keao Xieli Feed, Beijing, China) and water ad libitum were accessible. Animal beddings were replaced, and the cages were treated with high-pressure weekly to keep hygienic conditions. Inhalation of anesthetics with CO2 was used for the sacrifice of rats. Professor Shih-Hsin Lu synthesized NMBzA, and the purity was identified to be >98% by high-performance liquid chromatography/mass spectrometry. NMBzA was dissolved in DMSO and diluted with sterilized PBS and was injected subcutaneously at a final concentration of 0.35 mg/kg. The processing time was 8 weeks continuously, 3 times per week. Artesunate was dissolved by DMSO and suspended in diluted carboxymethylcellulose (Sigma–Aldrich, C5678), and was given by gavage at 10 mg/kg/day.

After sacrifice, rat esophagi were removed and opened longitudinally, the tumor numbers and sizes were counted, and the tissues were stored in formalin. Regular hematoxylin & eosin (H&E) staining and Immunohistochemistry (IHC) were performed as described previously.^19^ ^,^ ^56^ The procedure for defining “nuclear NRF2-positive cells” refers to our previous study.^19^ In detail, NRF2 nuclear expression level was determined by calculating the proportion of tumor cells with positive NRF2 nuclear staining among all viable tumor cells.

The primary antibodies used for IHC were as follows: NRF2 (Abcam, ab62352, 1:200), p53 (Proteintech, 60283-2-Ig, 1:1000), G6PD (Abcam, ab210702, 1:1000), SLC7A11 (Invitrogen, PA1-16893, 1:500), and NQO1 (Proteintech, 67240-1-Ig, 1:2000).

### Transcriptomic analysis (RNA sequencing)

The total RNA samples were extracted using Trizol (Invitrogen, 15596018) and examined for concentration and purity before sequencing. The Illumina NovaSeq 6000 platform was applied for gene sequencing. For the identification of differentially expressed genes, fold change > = 2 or < = 1/2 and FDR < 0.01 was used as screening standard. Gene Ontology (GO) enrichment was performed to investigate biological process differences, and Kyoto Encyclopedia of Genes and Genomes (KEGG) enrichment was performed for functional analysis.

### Statistical analysis

For multiple group comparisons, ANOVA analysis was applied. For other comparisons between 2 groups, we performed Student’s t-test. *p* < 0.05 was considered as statistically significant. Data were presented as mean ± SD regularly. We repeated the experiments 3 times biologically to confirm the results. GraphPad Prism 9.0 software was used for statistical analysis, curve-fitting, and statistical graph drawing. The Western blot band intensities were quantified via ImageJ and normalized to those of β‑actin for loading correction. Relative protein expression was calculated relative to the control group.

## Supplementary Material

figS4.tif figS4.tif

figS1.tif figS1.tif

figS5.tif figS5.tif

figS2.tif figS2.tif

figS6.tif figS6.tif

figS3.tif figS3.tif

Supplementary Material Supplementary Captions

## Data Availability

All data presented within the article and its Supplementary information files are available upon request from the corresponding author.
