# Supplementary material for: NRF2 and mutant p53 separately regulate redox balance in esophageal squamous cell carcinoma through metabolic reprogramming
Source: Cancer Biol Ther. 2026 Nov 6;27(1):2745287. doi: 10.1080/15384047.2026.2745287 (PMC13757581; doi:10.1080/15384047.2026.2745287)
Supplement: Supplementary file 1 [file KCBT_A_2745287_SM7827.docx]

**Figure S1.** CUL3-KEAP1-NFE2L2 axis gene variations in SCCs from TCGA database. (A-C) Genetic variation pattern and aberration rate of CUL3-KEAP1-NFE2L2 axis in ESCC group and ESCC+HNSCC+LUSC group. (D) Variations observed in CUL3 and the hot spot site found in the Cul3 Nedd8 domain. (E) High frequency of mutations found in KEAP1, mainly located in the BTB domain, the BACK domain and the Kelch domains. (F) Mutations explored in NRF2, which are distributed focus on N-terminus for KEAP1 interaction. ESCC, esophageal squamous cell carcinoma. HNSCC, head and neck squamous cell carcinoma. LUSC, lung squamous cell carcinoma.

**Figure S2.** Antioxidant effect of activated-NRF2. (A-B) Established NRF2 downstream gene mRNA expression discrepancy between CUL3-KEAP1-NFE2L2 axis-mutated and -normal groups in ESCC (A) and ESCC+HNSCC+LUSC (B) from TCGA database. All the listed genes encode proteins with antioxidant capacity. (C-D) ROS production variations under ART treatment at 2μM before and after KI696 treatment at 10μM in D3 cell. **** p<0.0001, ns non-significant. ART, artesunate. ROS, reactive oxygen species.

**Figure S3.** (A-B) G6PD and SLC7A11 mRNA expression discrepancy between CUL3-KEAP1-NFE2L2 axis-mutated and -normal groups in ESCC (A) and ESCC+HNSCC+LUSC (B) from TCGA database. (C-D) Knockdown efficacy of G6pd and Slc7a11 using RNAi at protein level in D3 cell. Based on the result, siSLC7A11-3 and siG6PD-2 were selected for further experiment. (E) RT-qPCR for canonical NRF2 downstream gene *Gclm*, *Sod1*, *Pgd*, *Tkt*, *Taldo1*. (F-G) The rescue effect of another antioxidant Trolox at 100μM for organoids derived from KYSE 180 and KYSE 450 under treatment of RRx-001 and HG106, with and without radiotherapy.**** p<0.0001, ns non-significant.

**Figure S4.** TP53 mutation induces DN and GOF effects in D3 cell. (A) A high mutation rate of almost 76% is observed in TP53 in ESCC patients from TCGA database. (B) Mutation patterns of TP53 in ESCC. Large of TP53 mutations are missense mutations and are intensively distributed in the DBD. (C) The plasmid constructure of Tp53^R246W^. (D) Immunoblots of DNA damage response system proteins after administration of DNA damage inducer Adriamycin at 0.2μM in D3-3xFLAG and D3- p53^R246W^ cell. This suggests p53^R246W^ mutant introduces the DN effect by impairing the DNA damage response system. (E) Cell viability variation between D3-3xFLAG and D3- p53^R246W^ cell. (F) Immunoblots of proliferation marker protein ERK1/2 in D3-3xFLAG and D3- p53^R246W^ cell. Fig. E-F indicates that p53^R246W^ mutant induces the GOF effect by activating cell proliferation signal in D3 cell. (G-H) Cell cycle analysis in D3-3xFLAG and D3- p53^R246W^ cell. * p<0.05, ns non-significant. DBD, DNA binding domain. DN, dominate negative. GOF, gain of function.

**Figure S5.** Reference genomic sequences of human SLC7A11 (GRCh38/hg38) and mouse Slc7a11 (GRCm38/mm10) were retrieved from the NCBI RefSeq database [67]. Taking the transcription start site (TSS) as the reference anchor, the 2000 bp upstream regulatory fragments of both genes were extracted for transcription factor binding site analysis. The human target region spanned chr4:138,162,097–138,164,097; the mouse regulatory interval covered chr3:50317385–50319385.

The position weight matrix (PWM) model for transcription factor Ets1 was obtained from the JASPAR 2026 database [68]. The human and mouse promoter sequences of SLC7A11/Slc7a11 were scanned for putative cis-regulatory elements using the built-in scanning tool of JASPAR 2026. A relative profile score threshold of ≥0.8 was set to screen high-confidence binding sites, and the relative position, genomic coordinates and core nucleotide sequence of each candidate binding site were recorded.

The integrated Sequence Logo module of JASPAR 2026 [68] was used to visualize the Ets1 binding motif based on information entropy; the vertical axis represented base conservation measured in bits to characterize the nucleotide preference of the Ets1 binding motif. All schematic diagrams and sequence logo plots presented in this work were assembled and illustrated via FigDraw.

**Figure S6.** Detailed information of animal experiment. (A) Schematic diagram of overall arrangement of rat model experiments. A stands for ART-only group, B stands for NMBzA-only group, C stands for ART-pretreated group, D stands for NMBzA+ART group. Thirty-two F344 rats were randomly divide into the 4 groups. Due stands for the end of every experiment stage and for the sacrifice of a portion of rats. (B) Typical pathological images of the rat model carcinogenesis course including normal, hyperplasia, dysplasia, papilloma and carcinoma. Scale bar 200μm for the left column and 50μm for the right column. (C) The dynamic body weight changes of rats in the 4 groups. (D) Quantification of NRF2 nuclear positive percents in the 4 groups. (E) Immuno-histochemistry of NRF2, p53, G6PD and SLC7A11 in the 4 groups. Scale bar 50μm. ** p<0.01, *** p<0.001. ART, artesunate. NMBzA, N-nitroso-N-methylbenzylamine.
